# Assessment of Aliasing Errors in Low-Degree Coefficients Inferred from GPS Data

**DOI:** 10.3390/s16050679

**Published:** 2016-05-11

**Authors:** Na Wei, Rongxin Fang

**Affiliations:** GNSS Research Center, Wuhan University, Wuhan 430079, Hubei, China; rfang@whu.edu.cn

**Keywords:** optimal truncation degree, scaled sensitivity matrix approach (SSM), simulation study

## Abstract

With sparse and uneven site distribution, Global Positioning System (GPS) data is just barely able to infer low-degree coefficients in the surface mass field. The unresolved higher-degree coefficients turn out to introduce aliasing errors into the estimates of low-degree coefficients. To reduce the aliasing errors, the optimal truncation degree should be employed. Using surface displacements simulated from loading models, we theoretically prove that the optimal truncation degree should be degree 6–7 for a GPS inversion and degree 20 for combing GPS and Ocean Bottom Pressure (OBP) with no additional regularization. The optimal truncation degree should be decreased to degree 4–5 for real GPS data. Additionally, we prove that a Scaled Sensitivity Matrix (SSM) approach can be used to quantify the aliasing errors due to any one or any combination of unresolved higher degrees, which is beneficial to identify the major error source from among all the unresolved higher degrees. Results show that the unresolved higher degrees lower than degree 20 are the major error source for global inversion. We also theoretically prove that the SSM approach can be used to mitigate the aliasing errors in a GPS inversion, if the neglected higher degrees are well known from other sources.

## 1. Introduction

The Global Positioning System (GPS) can provide the high-quality and long-term observations of surface displacements that are required for the study of surface mass redistributions, including the mass of atmosphere, ocean and continental water [1,2,3]. However, GPS site distribution over the land is sparse and uneven: few GPS stations are located at the Southern Hemisphere, and denser GPS stations are built in Europe and North America than the other continents. Also few GPS observations can be obtained over the ocean except for some stations located on small islands. Thus with a lack of observations, GPS data alone is just barely able to infer low-degree coefficients in the surface mass field. Therefore, previous studies usually truncate the estimated coefficients to low degrees in GPS-only inversions [4,5,6,7,8]. The unresolved higher-degree coefficients turn out to introduce aliasing errors into the low-degree coefficient estimates [9]. Additionally, most of the previous studies usually use various degrees of truncation and do not mention how these truncation degrees are determined [4,6].

In this paper, two important questions for inferring surface mass variations from GPS data are addressed: (1) the optimal truncation degree is investigated using simulated GPS displacements and (2) we quantify and mitigate the aliasing errors induced by the neglected higher degrees using a Scaled Sensitivity Matrix (SSM) approach. The structure of the paper is as follows: in Section 2, GPS surface displacements are simulated from the sum of atmospheric, oceanic and hydrologic models. In Section 3, the optimal truncation degree of the GPS inversion is determined by comparing the estimated coefficients with the reference coefficients integrated from loading models. In Section 4, we introduce the SSM approach, a quantitative approach for assessing the effects of unresolved parameters, to quantify and then reduce the aliasing errors of unresolved higher degrees [2].

## 2. GPS Surface Displacements

### 2.1. Simulated Data

The simulated GPS surface deformation is formed from the sum of atmospheric, oceanic and hydrologic models. For atmospheric and oceanic data, we directly used the monthly Atmosphere and Ocean De-aliasing Level-1B (AOD1B RL05) gravity coefficients up to degree 60 provided by Geo ForschungsZentrum (GFZ) [10]. AOD1B (RL05) coefficients are deduced from European Centre for Medium-Range Weather Forecasts (ECMWF) analysis data and the output from the baroclinic Ocean Model for circulation and tides (OMCT). AOD1B is used for the correction of short-term non-tidal atmospheric and oceanic mass variations for GRACE gravity products. We used the hydrologic data derived from the NOAH 2.7.1 land hydrology model in the Global Land Data Assimilation System (GLDAS) [11]. The monthly averaged snow and soil moisture is provided on a grid of 1.0° × 1.0° in form of equivalent water height from 2003 to 2010.

We first derive the spherical coefficients up to degree and order 60 (in terms of equivalent water) by integrating GLDAS grids following [12]. Then we calculate the GPS surface deformation from integrating both the AOD1B and GLDAS spherical coefficients up to degree and order 60 following [13]. No noise is simulated into the GPS displacements. The sum of AOD1B and GLDAS spherical coefficients are used as reference values hereafter to investigate the optimal truncation degree and assess the aliasing errors of unresolved higher-degree coefficients.

### 2.2. Real Data

The real GPS data used are the GPS residual time series provided by the Institut Géographique National (IGN) as a by-product of ITRF2008 data processing. The International GNSS Service (IGS) submitted solutions for ITRF2008 are the first reprocessing of GPS data collected by the IGS global network since 1997, in a fully consistent way using the latest models and methodologies. Outliers and offsets have also been detected and removed. A total of 560 GPS stations are included in the GPS network spanning the time period from 1997.0 to 2009.5. For a more detailed discussion of the ITRF2008 products, the reader is referred to [14,15]. The weekly ITRF08-GPS residuals are averaged, in a weighted sense, into monthly epochs. The variance matrixes are also propagated to form monthly variances.

## 3. Determination of the Optimal Truncation Degree

### 3.1. GPS Inversion

According to the loading theory [13], the three-dimensional GPS displacements (east, north, and up) due to the surface mass loading can be expressed in a sum of normalized spherical harmonic coefficients of surface mass density ($\Delta T_{nm}^{C}$, $\Delta T_{nm}^{S}$):

(1) $$\begin{array}{l} \Delta E(\Omega )=\sum_{n=1}^{n_{t}}\sum_{m=0}^{n}\frac{3l_{n}^{\prime }}{\rho_{E}(2n+1)}\frac{m{\bar{P}}_{nm}(\text{sin}\;\varphi )}{\text{cos}\;\varphi }(-\Delta T_{nm}^{C}\text{sin}\;m\lambda +\Delta T_{nm}^{S}\text{cos}\;m\lambda )\\ \Delta N(\Omega )=\sum_{n=1}^{n_{t}}\sum_{m=0}^{n}\frac{3l_{n}^{\prime }}{\rho_{E}(2n+1)}\partial_{\varphi }{\bar{P}}_{nm}(\text{sin}\;\varphi )(\Delta T_{nm}^{C}\text{cos}\;m\lambda +\Delta T_{nm}^{S}\text{sin}\;m\lambda )\\ \Delta H(\Omega )=\sum_{n=1}^{n_{t}}\sum_{m=0}^{n}\frac{3h_{n}^{\prime }}{\rho_{E}(2n+1)}{\bar{P}}_{nm}(\text{sin}\;\varphi )(\Delta T_{nm}^{C}\text{cos}\;m\lambda +\Delta T_{nm}^{S}\text{sin}\;m\lambda ) \end{array}$$

where ϕ and λ are latitude and longitude. ρ_*E*_ is the mean density of the Earth (5514 kg/m^3^). $h_{n}^{\prime }$ and $l_{n}^{\prime }$ are the degree-n Love numbers [13]. *P_nm_* is the normalized associated Legendre polynomials.

**Figure 1:**
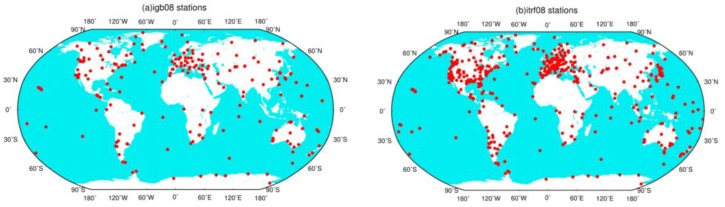
Two site distribution: 232 reference stations of IGb08 (**a**), and 560 reference stations of ITRF08 (**b**).

In this section, we use simulated GPS displacements to investigate the optimal truncation degree *n_t_* in Equation (1). To investigate the effects of various site distribution on the choice of the optimal truncation degree, two real site distribution are chosen to reflect the current real-data distribution of GPS stations. One is the 232 reference stations (‘sta232’) of IGb08 [16], and the other is the 560 reference stations (‘sta560’) of ITRF08 [14], shown in Figure 1. These GPS reference stations have good quality and long-term observations. GPS surface displacements for these two scenarios are simulated separately following Section 2.1.

**Figure 2:**
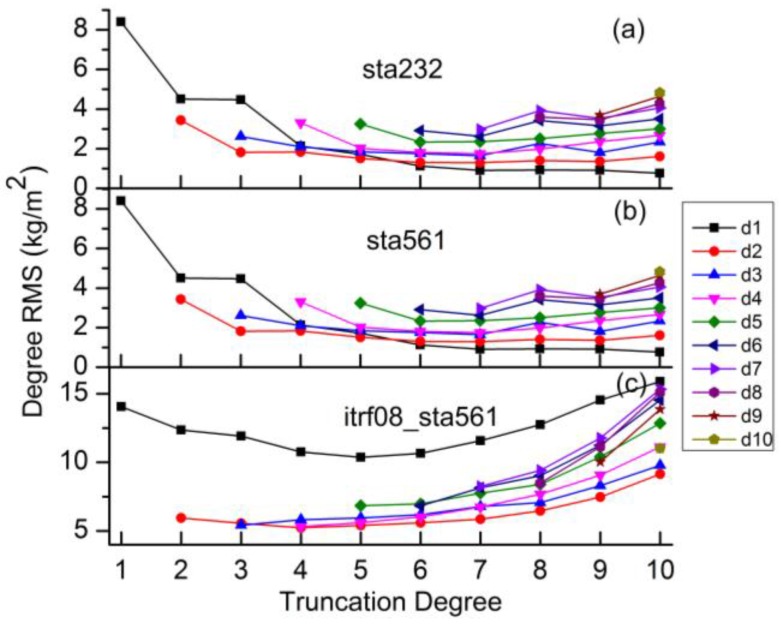
Degree RMS differences between coefficients inverted from GPS displacements and the reference values discussed in Section 2.1. The d1–d10 curves denote the degree RMS differences of degree 1-degree 10 coefficients inverted with different truncation degrees. Three scenarios of GPS displacements are used: The simulated displacements of 232 IGb08 stations (**a**) and 560 ITRF08 stations (**b**), and the real ITRF08-GPS residuals of 560 stations (**c**).

Then the simulated displacements are used to invert harmonic coefficients with various truncation degrees. The truncation degrees are ranged from degree 1 to degree 10 for both ‘sta232’ and ‘sta560’, as the normal equations would be singular for a much higher truncation degree. Figure 2 shows the degree Root Mean Square (RMS) differences between the estimated coefficients from simulated GPS inversion and the reference values (mentioned in Section 2.1). Results show that the degree RMS is prone to be stable (especially for degree-1 coefficients) when we estimate coefficients up to about degree 6. The optimal truncation degrees are degree 6 for ‘sta232’ and degree 7 for ‘sta560’, respectively. Comparisons between ‘sta232’ and ‘sta560’ indicate that higher degrees can be resolved and higher-quality estimates can therefore be derived with denser site distribution.

**Figure 3:**
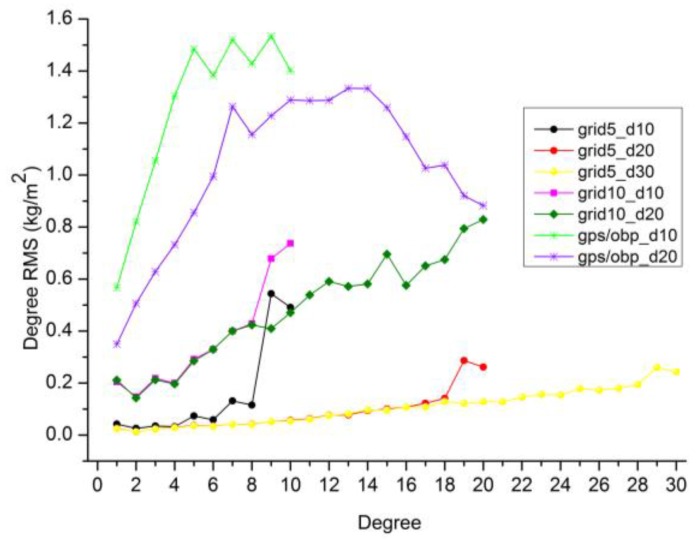
Degree RMS differences between coefficients inverted from various inversion schemes and reference values discussed in Section 2.1. Simulated data at global 5° × 5° (‘grid5’) and 10° × 10° (‘grid10’) grid points as well as simulated GPS/OBP (‘gps/obp’) are used. Different truncation degrees (‘d10’, ‘d20’ and ‘d30’) are tested for the three simulated scenarios.

We also simulate surface displacements on 5° × 5° and 10° × 10° global grids as the ideal cases for homogenous observations of surface displacements. Truncation degrees of degree 10, degree 20 and degree 30 are tested for the two global grids. The quality of harmonics coefficients inverted from homogenous grids are more than one order of magnitude better than those from real site distribution, by comparing the degree RMS in Figure 2 and Figure 3. For 5° × 5° grids, the quality of the estimated coefficients (except degree 19, 20) with a truncation degree of 20 is comparable to these with a truncation degree of 30. The reason for the larger degree RMS of degree 19/20 with the truncation degree of 20 (fringe effect) will be discussed in Section 4.3.

To investigate whether the above optimal truncation degrees derived from simulation studies are applicable to the real GPS data, we use ITRF08 residuals of 560 stations (‘itrf08_sta560’) mentioned in Section 2.2 to invert harmonic coefficients. These coefficients are then compared with the reference coefficients. Figure 2 shows that the optimal truncation degree is degree 4–5.

**Figure 4:**
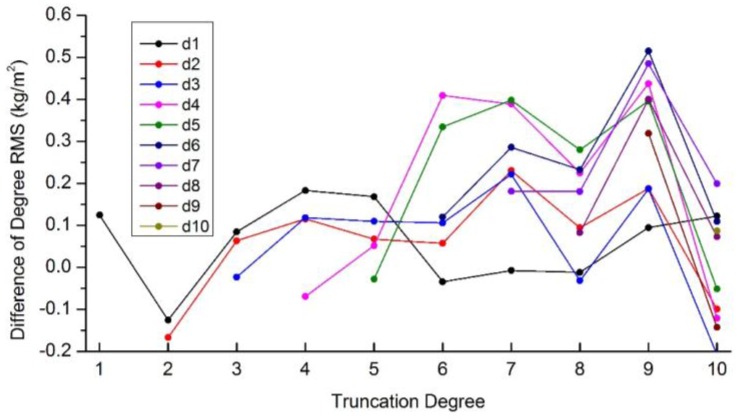
Degree RMS differences between coefficients inverted from the simulated displacements of 560 stations with and without data gaps and the reference values discussed in Section 2.1.

The optimal truncation degree of ‘itrf08_sta560’ is lower than ‘sta560’, which can be attributed to two potential reasons: (1) data gaps of ‘itrf08_sta560’ and (2) differences of the displacements observed by GPS and simulated by loading models. To analyze which is the major one, we first investigate the effects of data gaps involved in the real ITRF08-GPS residuals which are not considered for ‘sta560’. GPS displacements with the same data gaps as the real ITRF08-GPS data are simulated following Section 2.1. Results shows that degree 7 is still the optimal truncation degree for ‘sta560’ with data gaps (close to ‘sta560’ in Figure 2). And the degree RMS of ‘sta560’ with and without data gaps are very close (Figure 4). Data gaps have little effects on the determination of the optimal truncation degree. We therefore conclude the differences between the displacements observed by GPS and simulated by loading models are the major reason for the lower optimal truncation degrees of real GPS data.

### 3.2. GPS/OBP Inversion

Previous studies usually add OBP data to provide additional constraints of the mass variations over the ocean and therefore improve the quality of coefficients inferred from GPS observations [17,18,19]. However, we cannot estimate up to high enough degrees when no additional regularization is applied. Thus we still need to explore the optimal truncation degree for a GPS/OBP inversion in this case to improve the quality of the estimated coefficients as much as possible.

Simulated GPS displacements are referred to Section 2.1. As no original OMCT grid data are available, we use AOD1B (RL05) gravity coefficients to calculate the variations of OBP grids in form of equivalent water height over the ocean. Then the 6-hourly converted OBP grids are averaged to monthly data at a resolution of 5° × 5°. GPS and OBP data are combined in least square approach and the truncation degrees of degree 10 and degree 20 are tested here.

From Figure 3, we observe that the degree RMS of GPS/OBP inversion is unexpectedly larger than those of global grid inversion, although the number of observations of GPS/OBP are much more than 10° × 10° grids. One potential reason is that the complex combination of irregular GPS displacements over the land and some islands as well as homogenous grids (ocean bottom pressure) over the ocean in the GPS/OBP inversion. Some signals over the ocean may be enhanced and some signals over the land may be suppressed in such a complex combination.

Figure 3 also shows that the estimated coefficients with a truncation degree of 20 from the GPS/OBP inversion is obviously better than these with a truncation degree of 10, which is not the case for the homogenous grids. For homogenous grids, the quality of low degrees inverted with a lower truncation degree of 10 is close to those with a lower truncation degree of 20 (except fringe effect). The reason is that homogenous site distribution is more beneficial for recovering lower-degree coefficients, as homogenous site distribution can provide better long-wavelength signals and less short-wavelength signals than uneven site distribution. Thus a higher truncation degree is needed to resolve lower-degree coefficients from the uneven coverage of GPS and OBP observations.

### 3.3. Discussions

To obtain high-quality surface mass coefficients, we should balance between a higher truncation degree and a non-ill-conditioned normal equation. In case of a non-ill-conditioned normal equation, the effects of unresolved higher degrees on estimates are indeed reduced with a higher truncation degree. The condition of the normal equation depends on both the number of the observations and the spatial coverage of the site distribution. To obtain higher-quality estimates, denser and more homogenous site distribution should be chosen.

Considering the current status of IGS GPS site distribution and data length, for the GPS-only inversion, we suggest the truncation degree should be ranging from degree 4 to degree 7. For the GPS/OBP inversion, degree 20 is the reasonable truncation degree when no additional regularization is applied.

## 4. Assessment of Aliasing Errors Using SSM Approach

### 4.1. Introudction of SSM Approach

From Section 3, we know that better quality of harmonic coefficients can be derived from global inversion, if the optimal truncation degrees are used. However, the neglected higher-degree coefficients would still introduce errors into the estimates of low-degree coefficients. In this section, we use a SSM (Scaled Sensitivity Matrix) approach [2] to calculate and mitigate the aliasing errors of unresolved higher degrees. The SSM approach is a quantitative approach for assessing the effects of unresolved parameters. Chen, *et al.* [20] use the SSM approach to propose a simplified and unified model of multi-GNSS precise point positioning:

(2) $$L=\left[\begin{array}{cc} A_{1} & A_{2} \end{array}\right]\left[\begin{array}{c} X_{1}\\ X_{2} \end{array}\right]+\Delta$$

where *X*_1_ and *X*_2_ are the estimated low-degree coefficients and higher-degree coefficients, respectively. *A*_1_ and *A*_2_ are the design matrixes for the respective coefficients.

Then the normal equation can be written as follows:

(3) $$\left[\begin{array}{cc} N_{11} & N_{12}\\ N_{21} & N_{22} \end{array}\right]\left[\begin{array}{c} X_{1}\\ X_{2} \end{array}\right]=\left[\begin{array}{c} W_{1}\\ W_{2} \end{array}\right]$$

However, *X*_2_ cannot be directly or well resolved from *L* due to the sparse and uneven GPS site distribution. So *X*_2_ are usually neglected in the normal equation. The estimated coefficients should be truncated to about degree 4–7 according to the site distribution as discussed above. In this case the estimated ${\hat{X}}_{1}$ are:

(4) $${\hat{X}}_{1}=N_{11}^{-1}W_{1}$$

Replacing *W*_1_ in Equation (4) by Equation (3), the aliasing errors $\Delta {\hat{X}}_{1}$ introduced by unresolved *X*_2_ are obtained:

(5) $$\Delta {\hat{X}}_{1}=N_{11}^{-1}N_{12}X_{2}$$

$N_{11}^{-1}N_{12}$ is the scaled sensitivity matrix. Wu [9] use the scaled sensitive matrix to investigate the aliasing errors in geocenter motion. We here extend Wu’s study by using Equation (5) to determine the magnitude of aliasing errors of unresolved higher degrees in $\Delta {\hat{X}}_{1}$ providing that *X*_2_ is well known from external information.

### 4.2. Assessment of Aliasing Errors

**Figure 5:**
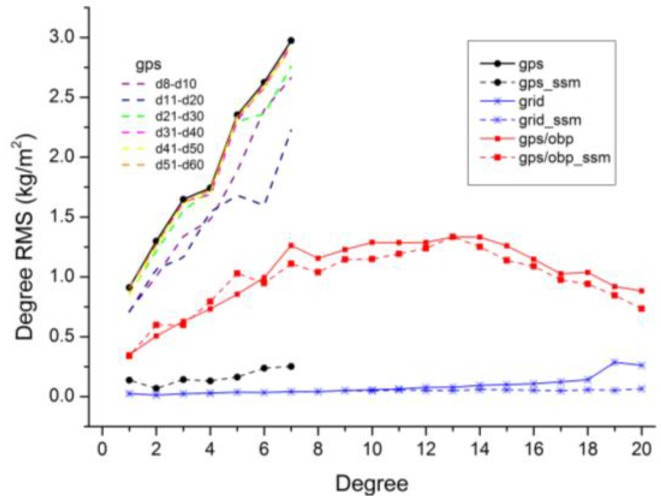
The degree RMS differences between low-degree estimates from various inversion schemes and reference values discussed in Section 2.1. Simulated GPS displacements (‘gps’), global 5° × 5° grids (‘grid’) and GPS/OBP (‘gps/obp’) are used. Solid lines and dashed lines denote the degree RMS differences before and after removing the aliasing errors, respectively. Dashed lines without symbols denote the degree RMS differences after removing the cumulative aliasing errors of d8–d10, d11–d20, d21–d30, d31–d40, d41–d50 and d51–d60 from GPS inversion, respectively.

**Figure 6:**
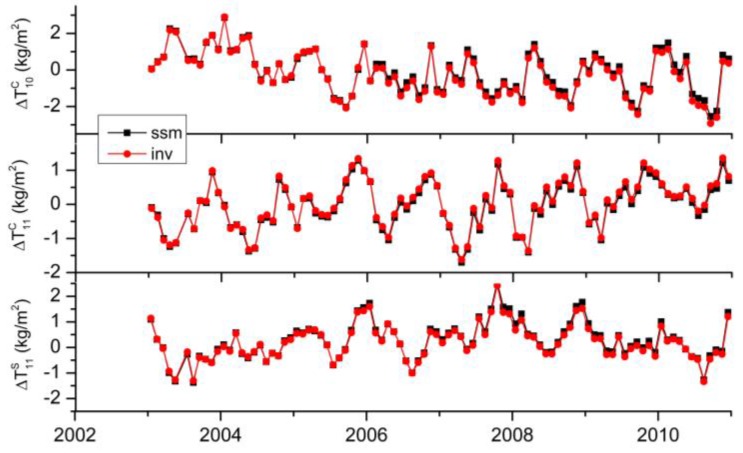
Aliasing errors of unresolved higher degrees in degree-1 coefficients calculated by the SSM approach (**black**); Differences between inverted degree-1 coefficients and the reference values (**red**). More than 99% of the ‘inv’ (**red**) variance is explained by ‘ssm’ (**black**).

We first use Equation (5) to calculate the aliasing errors in the simulated GPS inversion with a truncation degree of 7. The scaled sensitivity matrix is constructed using 560 stations. And the unresolved higher degrees are obtained from the reference values (mentioned in Section 2.1). From the degree RMS differences between the low-degree estimates and the reference values shown in Figure 5 , we find that the quality of estimated coefficients from the GPS inversion is significantly improved after removing the aliasing errors (black solid *vs.* black dashed). As an example, Figure 6 shows the time series of the aliasing errors in degree-1 terms (‘ssm’), and the differences between the inverted values and the reference values of degree-1 terms (‘inv’). We compute percentage of ‘inv’ variance explained by ‘ssm’, which are more than 99% for all the coefficients. In sum, we conclude that the unresolved higher degrees are the major error source for inverting low degrees. Other minor error sources may include the calculation errors and insufficient input data.

**Figure 7:**
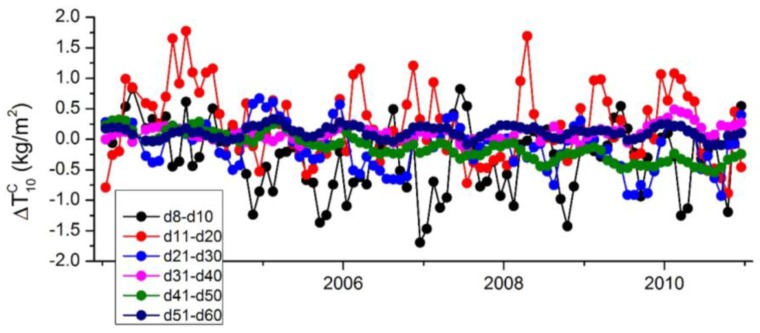
Aliasing errors of various higher degrees in $\Delta T_{10}^{C}$ calculated by the SSM approach.

Another important point is that the SSM approach can be used to calculate not only the sum of the aliasing errors of the unresolved higher degrees, but also the aliasing errors of any one or any combinations of the unresolved higher degrees. Thus the SSM approach is beneficial for us to identify the major error source from all the unresolved parameters. As an example, Figure 7 shows that the aliasing errors of various combinations of unresolved higher degrees in $\Delta T_{10}^{C}$ using the SSM approach. We observe that the major aliasing errors are caused by the unresolved degrees lower than degree 30 (especially lower than degree 20), while the higher degrees (degree 40–50) have little effects in $\Delta T_{10}^{C}$. Similar conclusions can be drawn for other low-degree estimates, indicated by the reduction of degree RMS after removing the cumulative aliasing errors of d8–d10, d11–d20, d21–d30, d31–d40, d41–d50 and d51–d60, respectively (Figure 5).

We also use the SSM approach to calculate and then remove the aliasing errors in the grid inversion and the GPS/OBP inversion truncated up to degree 20, shown in Figure 5. For 5° × 5° grid inversion, the improvement of estimates lower than degree 10 is limited, as the aliasing errors of unresolved higher degrees are small enough when we can estimate up to degree 20. Except for some low-degrees, certain improvements of estimates are observed after removing the aliasing errors in the GPS/OBP inversion.

From the above simulation studies, we theoretically conclude that the unresolved higher degrees, especially lower than degree 20, are the major error source for low-degree estimates. However, for real GPS data, experiments show that removing aliasing errors derived from the SSM approach cannot obviously improve the low-degree estimates (little improvement are still observed). One potential reason is the relatively larger amplitude of coefficients derived from real GPS data than from the loading models. Another potential reason is that the higher degrees (integrated from loading models) adopted to calculate the aliasing errors are not so consistent with these involved in real GPS observations, as the short-wavelength signals involved in loading models are smoothed. Thus, higher degrees that are more consistent with GPS observations should be known, if we want to improve low-degree estimates using SSM approach.

### 4.3. Discussionson Fringe Effects

**Figure 8:**
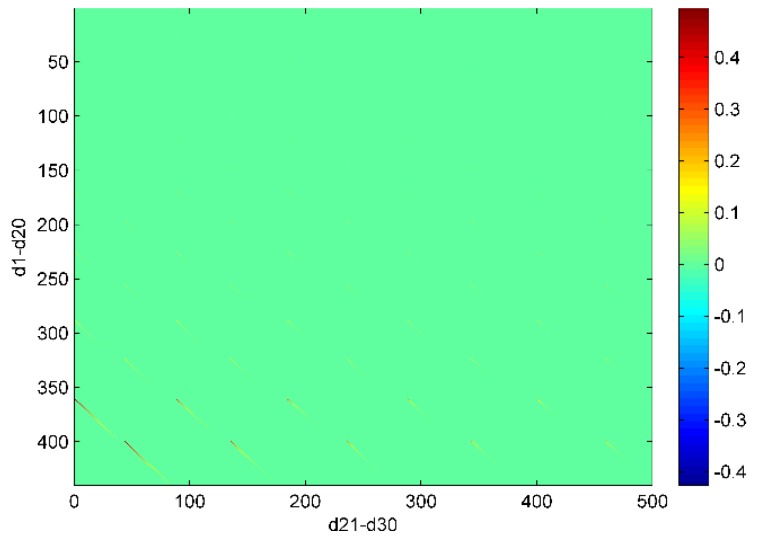
The scaled sensitivity matrix of 5° × 5° grid inversion with a truncation degree of 20. The horizontal axis is arranged in order of $\Delta T_{21,0}^{C},\;\Delta T_{21,1}^{C},\;\Delta T_{21,1}^{S},\;\ldots ,\;\Delta T_{30,30}^{C},\;\Delta T_{30,30}^{S}$ and the vertical axis is arranged in order of $\Delta T_{10}^{C},\;\Delta T_{11}^{C},\;\Delta T_{11}^{S},\;\ldots ,\;\Delta T_{20,20}^{C},\;\Delta T_{20,20}^{S}$ (from up to bottom).

We here use the scaled sensitivity matrix in Equation (5) to investigate why degree 19/20 from 5° × 5° grid inversion truncated up to degree 20 have larger RMS (fringe effects shown in Figure 3) than other estimated degrees. The scaled sensitivity matrix in fact represents the correlation of the unresolved higher degrees with the estimated degrees. High correlations are only observed between degree 19/20 and their adjacent unresolved higher degrees in Figure 8. So the larger degree RMS of degree 19/20 are caused by the adjacent unresolved higher degrees.

**Figure 9:**
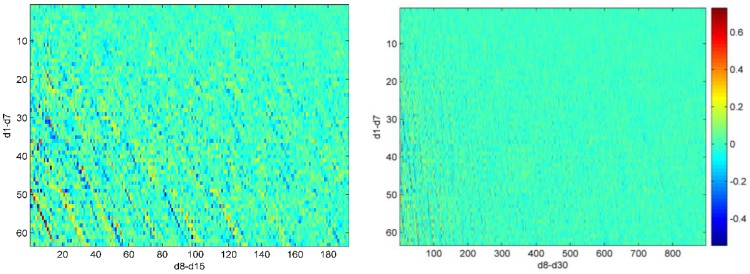
The scaled sensitivity matrix of simulated GPS inversion with a truncation degree of 7. The horizontal axis is arranged in order of $\Delta T_{80}^{C},\;\Delta T_{81}^{C},\;\Delta T_{81}^{S},\;\ldots ,\;\Delta T_{30,30}^{C},\;\Delta T_{30,30}^{S}$ and the vertical axis is arranged in order of $\Delta T_{10}^{C},\;\Delta T_{11}^{C},\;\Delta T_{11}^{S},\;\ldots ,\;\Delta T_{77}^{C},\;\Delta T_{77}^{S}$ (from up to bottom).

However, no obvious fringe effects are observed in GPS inversion shown in Figure 2. Although the correlations of degree 6/7 with the adjacent unresolved higher degrees are still higher, the correlations of degree 6/7 with other unresolved higher degrees and the correlation of degree 1–5 with the unresolved higher degrees are not negligible, shown in Figure 9. The effects of the unresolved higher degree adjacent to the truncation degree are not the only major error source in this case, so no obvious fringe effects are observed. In sum, if we can estimate up to high degrees (e.g., degree 20), the aliasing errors of the unresolved higher degrees on all estimates are generally suppressed and therefore the fringe effects would be highlighted.

## 5. Conclusions

Using surface displacements simulated from loading models, two important questions for inferring surface mass variations from GPS data are answered in this paper: (1) the optimal truncation degree and (2) the magnitude and mitigation of the aliasing errors induced by the unresolved higher degrees.

Using the SSM approach, we have demonstrated that the unresolved degrees lower than degree 30 (especially degree 20) are the major error source for the lower-degree estimates. Thus the aliasing errors can be effectively suppressed and good quality coefficients can be derived from global inversion truncated up to degree 20, considering that the current data coverage is not enough to estimate up to degree 30. This confirms the conclusions of the optimal truncation degrees for homogenous grids or GPS/OBP in Section 3. However, global inversion with data provided by sparsely and unevenly distributed GPS stations should be truncated to degree 4–7 to ensure the condition of the normal equation. Additionally, dense and even site distribution help to derive better quality of estimates.

From the scaled sensitivity matrix, we find the high correlations between the adjacent degrees which induces the so-called fringe effects. Therefore, another important thing for choosing the optimal truncation degree is that the truncation degree should not be close to the interested degrees to avoid the fringe effects.

We also have theoretically proved that the SSM approach can be used to mitigate the aliasing errors in the GPS inversion with low truncation degrees, if the neglected higher degrees are well known from other sources. However, due to the inconsistencies of the middle-high degrees between GPS and loading models as well as GRACE [21], we currently cannot use the SSM approach to improve the low-degree estimates inferred from real GPS observations. However, the SSM approach is still a potential way to improve the estimates from the GPS inversion in the future, provided the consistency of the middle-high degree signals between GPS and other sources are improved.
